# Porcine rotavirus mainly infects primary porcine enterocytes at the basolateral surface

**DOI:** 10.1186/s13567-019-0728-x

**Published:** 2019-12-19

**Authors:** Tingting Cui, Sebastiaan Theuns, Jiexiong Xie, Hans J. Nauwynck

**Affiliations:** Department of Virology, Parasitology and Immunology, Faculty of Veterinary Medicine, Ghent University, Salisburylaan 133, 9820 Merelbeke, Belgium

## Abstract

Intestinal epithelium functions as a barrier to protect multicellular organisms from the outside world. It consists of epithelial cells closely connected by intercellular junctions, selective gates which control paracellular diffusion of solutes, ions and macromolecules across the epithelium and keep out pathogens. Rotavirus is one of the major enteric viruses causing severe diarrhea in humans and animals. It specifically infects the enterocytes on villi of small intestines. The polarity of rotavirus replication in their target enterocytes and the role of intestinal epithelial integrity were examined in the present study. Treatment with EGTA, a drug that chelates calcium and disrupts the intercellular junctions, (i) significantly enhanced the infection of rotavirus in primary enterocytes, (ii) increased the binding of rotavirus to enterocytes, but (iii) considerably blocked internalization of rotavirus. After internalization, rotavirus was resistant to EGTA treatment. To investigate the polarity of rotavirus infection, the primary enterocytes were cultured in a transwell system and infected with rotavirus at either the apical or the basolateral surface. Rotavirus preferentially infected enterocytes at the basolateral surface. Restriction of infection through apical inoculation was overcome by EGTA treatment. Overall, our findings demonstrate that integrity of the intestinal epithelium is crucial in the host’s innate defense against rotavirus infection. In addition, the intercellular receptor is located basolaterally and disruption of intercellular junctions facilitates the binding of rotavirus to their receptor at the basolateral surface.

## Introduction

Diarrhea is one of the most important causes of death in young piglets and can be evoked by viruses, bacteria and parasites. Rotaviruses are considered as the most important pathogens that cause diarrhea in piglets and children. They belong to the genus rotavirus within the family *Reoviridae*. It consists of a triple-layered capsid encapsulating a genome consisting of eleven segments of double-stranded RNA (dsRNA) that encodes six structural (VP1–VP4, VP6 and VP7) and six non-structural (NSP1–NSP6) proteins. Each gene segment encodes one viral protein, except for gene segment eleven which encodes NSP5 and NSP6. In vivo, rotavirus infects primarily mature enterocytes in the polarized intestinal epithelium. Many studies on rotavirus biology have been conducted in nonpolarized cultures of MA104 cells (monkey kidney epithelial cells), or intestinal epithelial cell lines IPEC-J2 and Caco-2 cells, since they are most permissive for replication of some genotypes of rotavirus strains [1–5]. However, there are still a lot of rotavirus strains that do not grow in these cells [6, 7]. Therefore, it is necessary to have a culture system of intestinal enterocytes for the investigation of rotavirus replication mechanisms.

Attachment and entry into the target cells are the first steps of viral infection, and several cell surface molecules have been identified as receptors or co-receptors for rotaviruses, such as sialic acids, integrins and hsc70 using continuous cell lines [8–11]. More recently, histo-blood group antigens have been reported to mediate the attachment of rotavirus strains [12–14]. Besides, the junction proteins JAM-A, occludin and ZO-1 have also been suggested to play an important role during rotavirus entry into MA104 cells [15, 16].

Tight junctions are essential for establishing a barrier between different compartments of the body. Their primary physiological role is to function as paracellular gates which restrict diffusion on the basis of size and charge. The main protein components of the transmembrane strands are proteins of the claudin family and three junctional marvel domain proteins: occludin, tricellulin and MarvelD3 [17]. The junctional adhesion molecules (JAMs) and zonula occludens (ZO) are also important proteins of tight junctions. Tight junction components are known to be targets for various pathogenic bacteria and viruses. They hijack the recycling process of tight junction proteins (internalization, hydrolysis, synthesis, and egression) to enter and infect cells or target junctional signaling mechanisms to open the intercellular space. For hepatitis C virus (HCV) entry, claudin-1 was identified as a key factor and claudin-6 and claudin-9 as additional co-receptors [18, 19]. JAM-A with two extracellular V-type Ig domains serves as a receptor for feline calicivirus and all three serotypes of reovirus [20, 21]. Polarized epithelial cells are characterized by the presence of two distinct plasma membrane domains: the apical, corresponding to the luminal surface, and the basolateral, corresponding to the serosal surface [22, 23]. The two domains are separated by tight junctions which play an important role in maintaining the cell polarity and the unique protein-lipid composition of each domain. The best characterized experimental epithelial cell system for the study of polarized virus infection is that of the Madin-Darby canine kidney (MDCK) cell [24, 25] which provides a convenient model for the study of epithelial cell polarity. The entry of vesicular stomatitis virus (VSV), type C retrovirus, human cytomegalovirus (HCMC), Semlike Forest virus (SFV), equine herpesvirus-1 (EHV-1) and bovine herpesvirus-4 (BoHV-4) occurs at the basolateral membrane of polarized epithelial cells [26–30], whereas simian virus 40 (SV40), respiratory syncytial virus, hepatitis A virus (HAV) preferentially infect cells at the apical surface [31, 32].

Most studies of the rotavirus replication have been performed with MA104 cells, a poorly differentiated monkey kidney epithelial cell line. These cells do not exhibit the highly polarized and differentiated phenotype of enterocytes in vivo. The human intestinal epithelial cell line Caco-2, established from a human colon adenocarcinoma, has been shown to spontaneously display the morphologic and biochemical properties of mature enterocytes [33]. Caco-2 cells are polarized with two plasma membrane domains: an apical one which faces the external lumen and a basolateral one which faces the internal milieu. Svensson et al. demonstrated that the MA104-cell adapted rhesus rotavirus (RRV) could infect both the apical and basolateral domains of Caco-2 cells in a symmetric manner, whereas the virions were secreted preferentially from the apical plasma membrane [34, 35]. In another study, Ciarlet et al. demonstrated that NA-sensitive strains infect efficiently only from the apical surface, NA-resistant strains infect equally well from the apical and basolateral surface [36]. Bugarcic and Taylor further demonstrated that the NSP4, a nonstructural glycoprotein encoded by rotavirus which induces diarrhea by acting as an enterotoxin, was actively secreted into the culture medium preferentially from the apical surface of infected Caco-2 cells [37]. Rotavirus mainly infects the mature villus enterocytes of jejunum and ileum in vivo. The importance of polarization and intercellular junctions have been less examined for porcine rotaviruses in their target cells [36, 38]. In this study, the co-culture system of porcine enterocytes that was established in our laboratory was used for this study [39].

## Materials and methods

### Piglets and virus samples

Primary porcine enterocytes were isolated from the ileum of 3 days old piglets and co-cultured with porcine myofibroblasts [39]. The enterocytes were maintained with Dulbecco’s modified Eagle’s F12 Ham medium (DMEM-F12). Rotavirus strains, originating from porcine fecal samples, 12R050 (G5P[7]) and 12R046 (G9P[23]) were passaged 4 times on MA104 cells and have previously been shown to replicate in porcine primary enterocytes [6, 39, 40].

### Disruption of intercellular junctions

The enterocytes were grown in transwells or normal plastic wells with myofibroblasts. Cells were grown to confluency and the trans-epithelial electrical resistance (TEER) was measured until a steady TEER of ~500–700 Ω cm^−2^ was attained. TEER was measured prior to and following treatment with PBS containing 8 mM ethylene glycol tetra-acetic acid (EGTA) (VWR International, Leuven, Belgium) for 30 min at 37 °C. PBS alone was used as control. After 30 min, the cells were washed 3 times with DMEM. The viability of the cells was assessed by ethidium monoazide bromide (EMA) staining after an additional 24 h incubation.

### Replication kinetics of rotavirus in primary enterocytes treated with EGTA

After 2 days of cultivation, enterocytes were treated with PBS containing 8 mM EGTA or PBS alone for 30 min. Afterwards, cells were inoculated with trypsin-treated (5 µg/mL, 30 min at 37 °C) rotavirus 12R050 at a multiplicity of infection (MOI) of 0.05. Cells were washed 3 times with DMEM after 1 h of inoculation. Fresh culture medium containing 1 µg/mL trypsin was added to each well and the cells were further incubated at 37 °C and 5% CO_2_. At different time points (0, 9, 18 and 27 h) post-inoculation, the supernatant was collected for virus titration and cells were fixed with 4% paraformaldehyde for immunofluorescence staining.

### Rotavirus infection in primary enterocytes through different inoculation routes

The ability of rotavirus to infect enterocytes from the apical and the basolateral side was compared using enterocytes grown in a transwell system. After 2 days of cultivation, a monolayer of enterocytes was treated with PBS containing 8 mM EGTA or PBS alone. Next, cells were inoculated with rotavirus 12R050 and 12R046 at a MOI of 0.05 either at apical or basolateral surface for 30 min at 37 °C. Non-adsorbed virus particles were removed by washing 3 times with DMEM. Fresh culture medium containing 1 µg/mL trypsin was added to each well and cells were further incubated at 37 °C and 5% CO_2_. Nine hours post-inoculation, the supernatant was collected for virus titration and the cells were fixed with 4% paraformaldehyde for immunofluorescence staining.

### Viral binding, internalization and release assays

For the binding assay, after treatment with PBS containing 8 mM EGTA or PBS alone for 30 min, the cells were chilled on ice for 5 min and washed 3 times with DMEM. Cells were inoculated apically with rotavirus 12R050 and 12R046 at a MOI of 0.1 for 1 h at 4 °C and washed 3 times with DMEM to remove unbound particles. Cells were further incubated with medium for another 9 h at 37 °C and fixed for immunofluorescence staining.

For the internalization assay, cells were inoculated apically with rotavirus 12R050 and 12R046 at a MOI of 0.1 for 1 h at 4 °C. After virus binding to the cell surface, cells were washed 3 times with DMEM and then treated with EGTA or PBS for 30 min. After 3 washes with DMEM, cells were incubated with medium for 1 h at 37 °C. Afterwards, cells were washed 3 times with DMEM and further incubated at 37 °C for another 9 h.

For the release assay, cells were inoculated apically with rotavirus 12R050 and 12R046 at a MOI of 0.1 for 1 h at 37 °C. Then, cells were treated with EGTA or PBS for 30 min. After 3 washes with DMEM, cells were further incubated at 37 °C for another 9 h. The supernatants were harvested at 9 hpi for virus titration.

### Immunofluorescence staining and confocal microscopy

Double immunofluorescence stainings against rotavirus A antigens and cytokeratin were performed to specifically visualize the infected enterocytes. The cells were incubated with polyclonal guinea pig anti-monkey rotavirus SA-11 VP6 antibodies (kindly provided by John Patton) and 10% normal goat serum for 1 h at 37 °C, followed by goat anti-guinea pig-IgG FITC labelled antibodies (Southern Biotech) for 1 h at 37 °C [41]. Afterwards, cells were incubated for 1 h at 37 °C with monoclonal anti-human cytokeratin antibodies, followed by 1 h at 37 °C with goat anti-mouse-IgG Texas Red labelled antibodies (Molecular Probes). Nuclei were stained with Hoechst for 10 min at RT. Infected enterocytes were visualized by fluorescence microscopy.

### Virus titration

Rotavirus titration was conducted in MA104 cells grown confluently in 96-well plates. Ten-fold serial dilutions of supernatant (10^−1^–10^−8^) were inoculated and after 5 days the cytopathogenic effect (CPE) was examined using a light microscope. The cell culture infective dose (TCID_50_) was calculated using the formula of Reed and Muench [42].

### Neuraminidase treatment of enterocytes

To remove SAs from cells, monolayers of co-cultured enterocytes were washed 3 times with warm DMEM (MA104 cells were used as control and underwent the same manipulations). Then, cells were incubated with 50 mU/mL NA from *Vibrio cholerae* (Roche Diagnostics) at 37 °C for 1 h. Cells that were mock treated were incubated with DMEM and underwent the same manipulations as NA-treated cells. Afterwards, cells were inoculated with rotavirus 12R050 and 12R046 at a MOI of 0.1. After 60 min of incubation at 37 °C, cells were washed 3 times with DMEM and further incubated for 12 h (37 °C, 5% CO_2_). Then, cells were fixed with 4% paraformaldehyde for immunofluorescence staining.

### Statistical analysis

Data were statistically processed by GraphPad Prism 5.0 (GraphPad software, Inc., San Diego, CA, USA) for analysis of variance (ANOVA). The data are represented as means with standard deviation (SD) of three independent experiments. Results with *p* values of < 0.05 were considered significant.

## Results

### Viability of enterocytes after EGTA treatment

**Figure 1:**
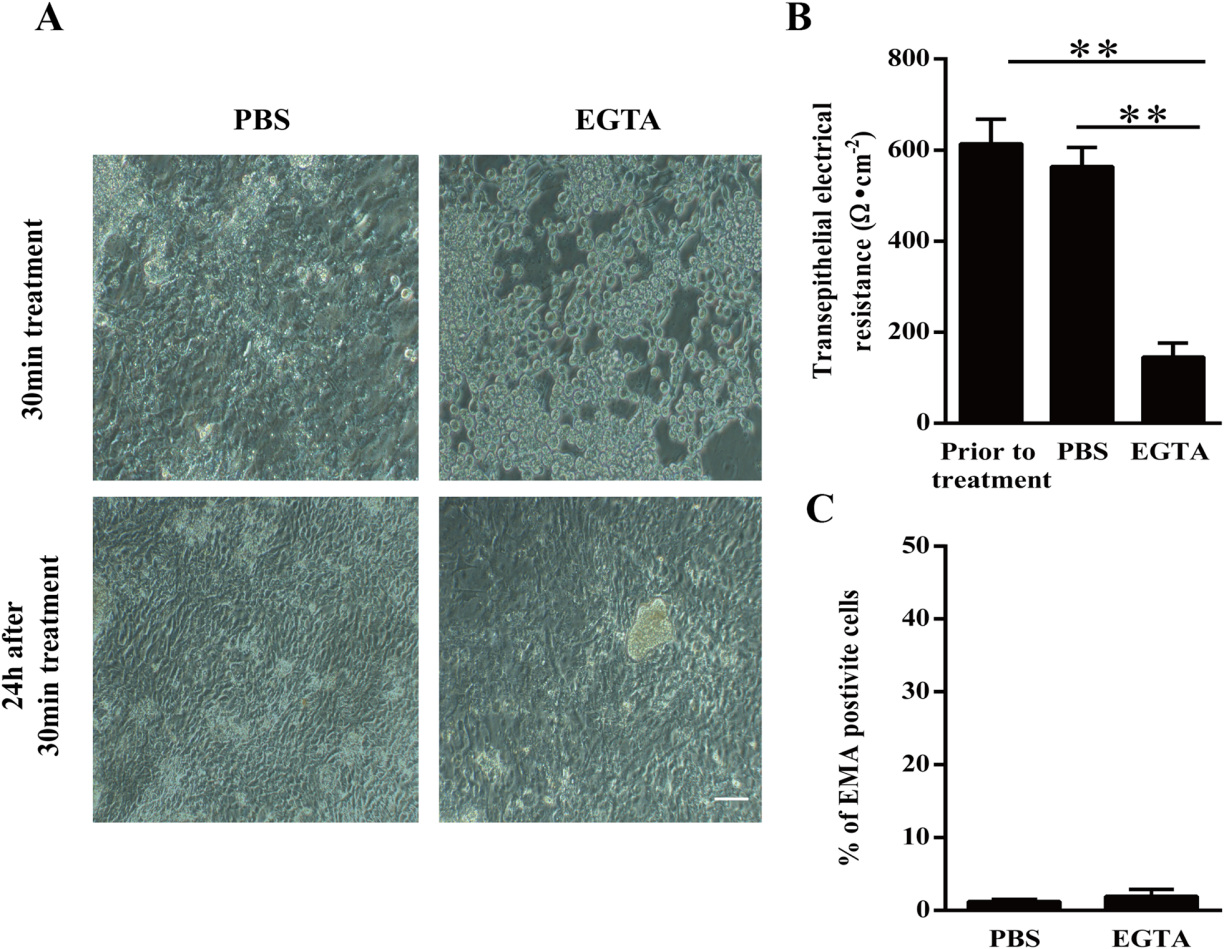
**Disruption and restoration of intercellular junctions in primary enterocytes after EGTA treatment. A** Representative microscopic images of enterocytes directly and 24 h after a 30 min treatment with EGTA. Scale bar: 100 µm. **B** Trans-epithelial electrical resistance of cells prior to treatment and after a 30-min treatment with PBS (control) or EGTA. **C** The percentage of EMA positive cells 24 h after treatment with PBS or EGTA. Data are expressed as the mean ± SD of the results of three separate experiments. Statistically significant (*p* < 0.01) differences are indicated with two asterisks.

After a 30-min treatment with PBS containing 8 mM EGTA, the enterocytes became round and lost their intercellular junctions (Figure 1A). The TEER significantly dropped to baseline levels after treatment with EGTA, but not after treatment with PBS. Twenty-four hours after EGTA treatment, the enterocytes formed again a monolayer with a stable TEER of 500–700 Ω cm^−2^ (Figure 1B), and only 2 ± 0.9% of cells were EMA positive, which means that the effect of EGTA was reversible and did not alter the cell viability (Figure 1C). These results show that enterocytes are able to restore their intercellular bridges upon addition of bivalent ions.

### Disruption of intercellular junctions between enterocytes increases their susceptibility to rotavirus

**Figure 2:**
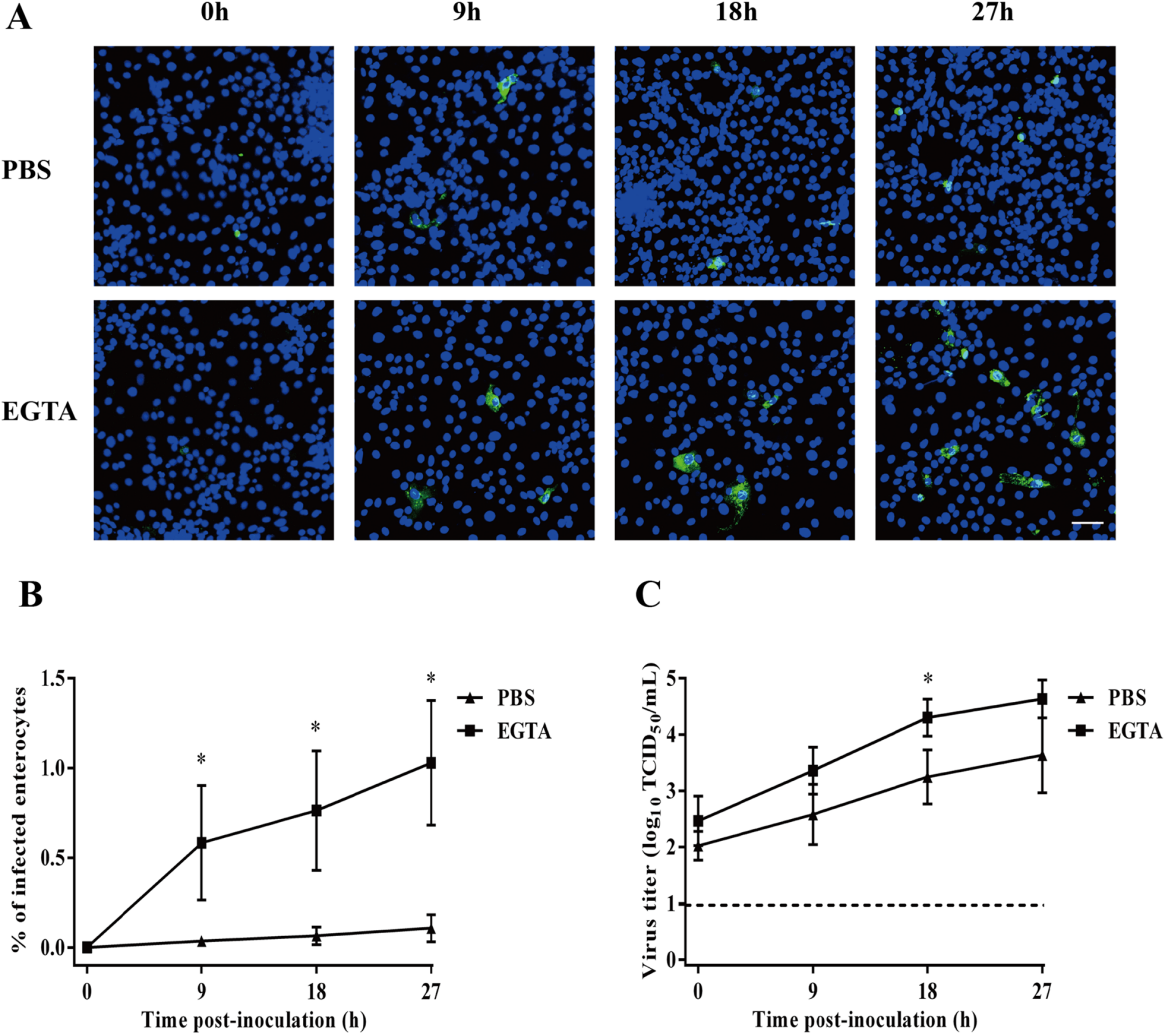
**Kinetics of rotavirus 12R050 (G5P[7]) replication in enterocytes after treatment with EGTA. A** Representative confocal images of rotavirus infection (green) in primary enterocytes after a 30-min treatment with EGTA or PBS at 0, 9, 18 and 72 hpi. Scale bar: 50 µm. **B** The percentage of rotavirus infected enterocytes was analyzed by immunofluorescence staining. **C** Virus titer was determined in the supernatant with MA104 cells. Data are expressed as the mean ± SD of the results of three separate experiments. Statistically significant (*p* < 0.05) differences are indicated with an asterisk.

The percentage of infection and the virus titer were evaluated at different time points post-inoculation (0, 9, 18 and 27 hpi) in EGTA or PBS treated cells. The percentage of infection was 0.001 ± 0.001 at 0 hpi, 0.04 ± 0.02% at 9 hpi, 0.07 ± 0.05% at 18 hpi and 0.1 ± 0.07% at 27 hpi after mock treatment with PBS. When the cells were treated with EGTA, the infection was significantly increased to 0.002 ± 0.0005% at 0 hpi, 0.6 ± 0.3% at 9 hpi, 0.8 ± 0.3% at 18 hpi and 1 ± 0.3% at 27 hpi (Figure 2B). The virus titer increased over time. After mock treatment with PBS, the virus titer was 10^2.0 ± 0.3^ TCID_50_/mL at 0 hpi, 10^2.6 ± 0.5^ TCID_50_/mL at 9 hpi, 10^3.2 ± 0.5^ TCID_50_/mL at 18 hpi and 10^3.6 ± 0.7^ TCID_50_/mL at 27 hpi. The virus titer was significantly higher in EGTA treated cells with a virus titer of 10^2.5 ± 0.4^ TCID_50_/mL at 0 hpi, 10^3.4 ± 0.4^ TCID_50_/mL at 9 hpi, 10^4.3 ± 0.3^ TCID_50_/mL at 18 hpi and 10^4.6 ± 0.3^ TCID_50_/mL at 27 hpi (Figure 2C). These results demonstrate that disruption of intercellular junctions clearly increases the susceptibility of primary enterocytes to rotavirus infection.

### Rotavirus preferentially infects at the basolateral surface of enterocytes

**Figure 3:**
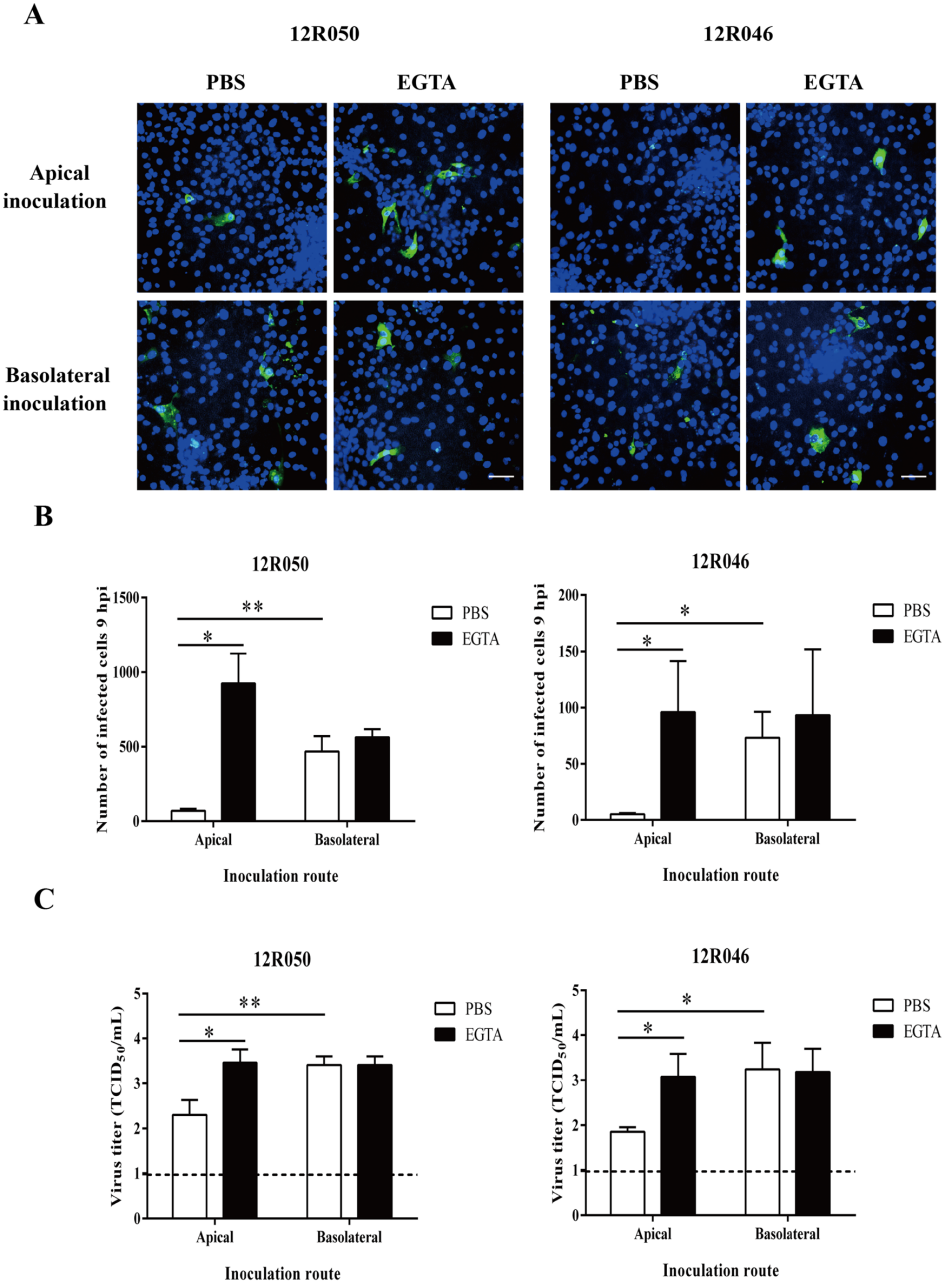
**Rotavirus preferentially infects the basolateral surface of enterocytes and disruption of ICJ overcomes the restriction of rotavirus infection at the apical surface. A** Representative confocal images of rotavirus infection (green) in enterocytes. Scale bar: 50 µm. To compare epithelial cell susceptibility to rotavirus, cells were exposed at either the apical surface or the basolateral surface to rotavirus 12R050 (G5P[7]) and 12R046 (G9P[23]). **B** The total number of infected cells per well was counted for each condition. **C** The virus titer was determined in supernatant for each condition. Data are expressed as the mean ± SD of the results of three separate experiments. Statistically significant differences are indicated with one asterisk (*p* < 0.05) and two asterisks (*p* < 0.01).

To investigate whether rotavirus preferentially infects enterocytes, either at the apical or the basolateral surface, we inoculated the cells with rotavirus 12R050 and 12R046 (MOI of 0.05) by both routes. Nine hours post-inoculation, the supernatant was collected for virus titration and the cells were fixed for immunofluorescence staining. Upon inoculation with rotavirus 12R050 and 12R046, 8 and 15 times more cells per well were infected upon basolateral inoculation than upon apical inoculation. To determine whether intercellular junctions also protect the basolateral surfaces of enterocytes from rotavirus infection, cells were treated with 8 mM EGTA before inoculation at apical (and basolateral as control) surfaces. Disruption of intercellular junctions increased the infection upon inoculation with 12R050 and 12R046 at the apical surface with a factor of 13 and 19, respectively. Treatment with EGTA did not change infection after inoculation at the basolateral surfaces with neither of the two strains (Figure 3B). The virus titer was significantly higher upon basolateral inoculation (10^3.4 ±^ ^0.2^ TCID_50_/mL for 12R050 and 10^3.2 ± 0.6^ TCID_50_/mL for 12R046) than upon apical inoculation (10^2.3 ± 0.3^ TCID_50_/mL for 12R050 and 10^1.9 ± 0.1^ TCID_50_/mL for 12R046) (Figure 3C).

### Increased susceptibility of enterocytes to rotavirus infection is correlated with the virus entry step

**Figure 4:**
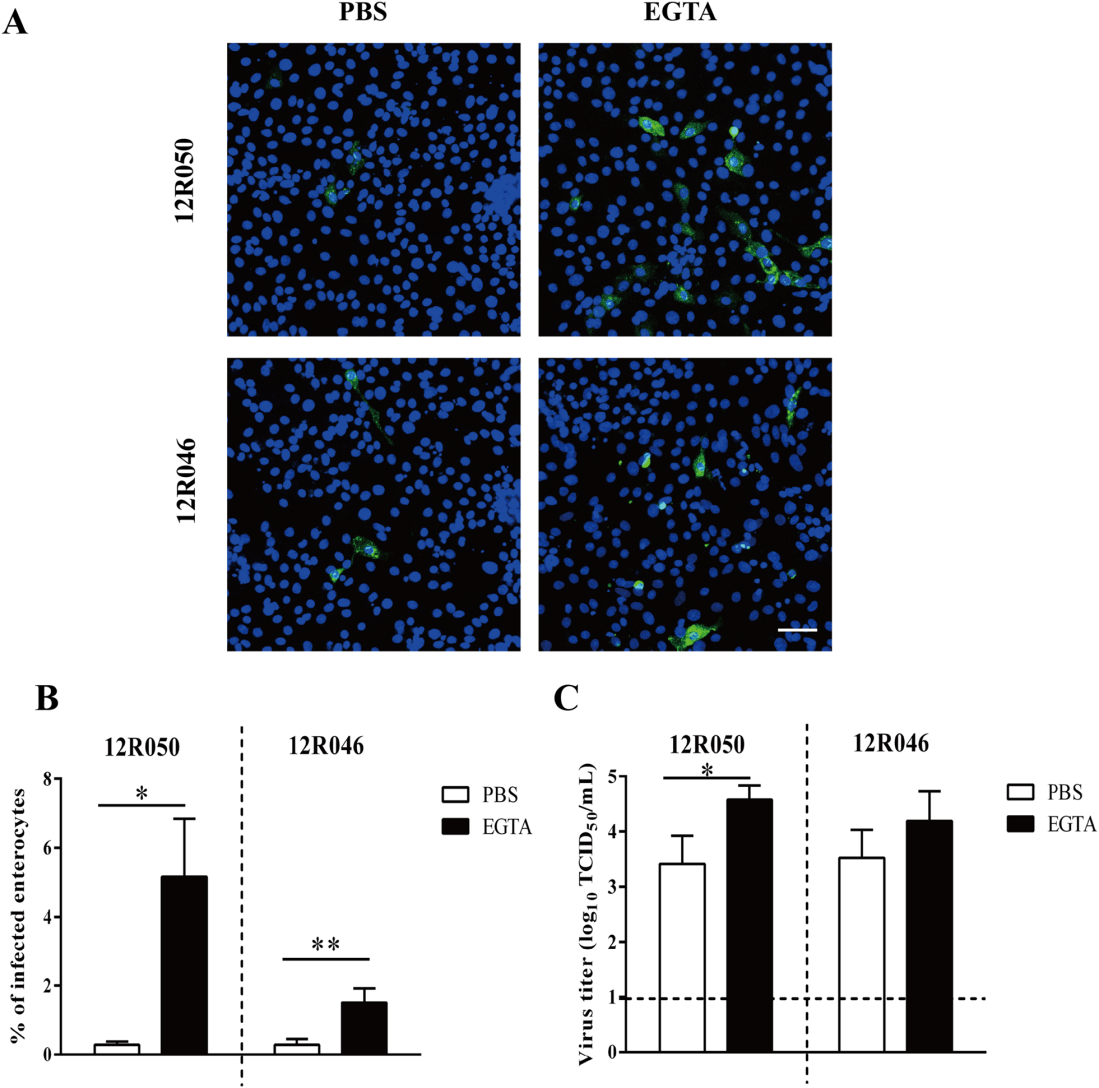
**EGTA treatment increases the binding of rotavirus to enterocytes.** Cells were pre-incubated with PBS or EGTA and inoculated with rotavirus at 4 °C for 1 h. After removing the unbound viral particles, cells were further incubated for 9 h. **A** Representative confocal image of infection in enterocytes. Scale bar: 50 µm. The percentage of infected cells (**B**) and the virus titer (**C**) were determined. Data are expressed as the mean ± SD of the results of three separate experiments. Statistically significant differences are indicated with one asterisk (*p* < 0.05) and two asterisks (*p* < 0.01).

We first examined rotavirus binding to enterocytes after an EGTA treatment. After 30 min treatment with EGTA, enterocytes were inoculated apically with rotavirus 12R050 and 12R046 (MOI of 0.1) at 4 °C for 1 h. The percentage of infection and virus titer were determined at 9 hpi. After EGTA treatment, the percentage of infection was 18 and 5 times higher than after mock PBS treatment for 12R050 and 12R046, respectively. The virus titer after EGTA treatment (10^4.6^ ^± 0.3^ TCID_50_/mL for 12R050 and 10^4.2^ ^± 0.5^ TCID_50_/mL for 12R046) was higher than after mock PBS treatment (10^3.4 ± 0.5^ TCID_50_/mL for 12R050 and 10^3.5^ ^± 0.5^ TCID_50_/mL for 12R046) (Figure 4).

**Figure 5:**
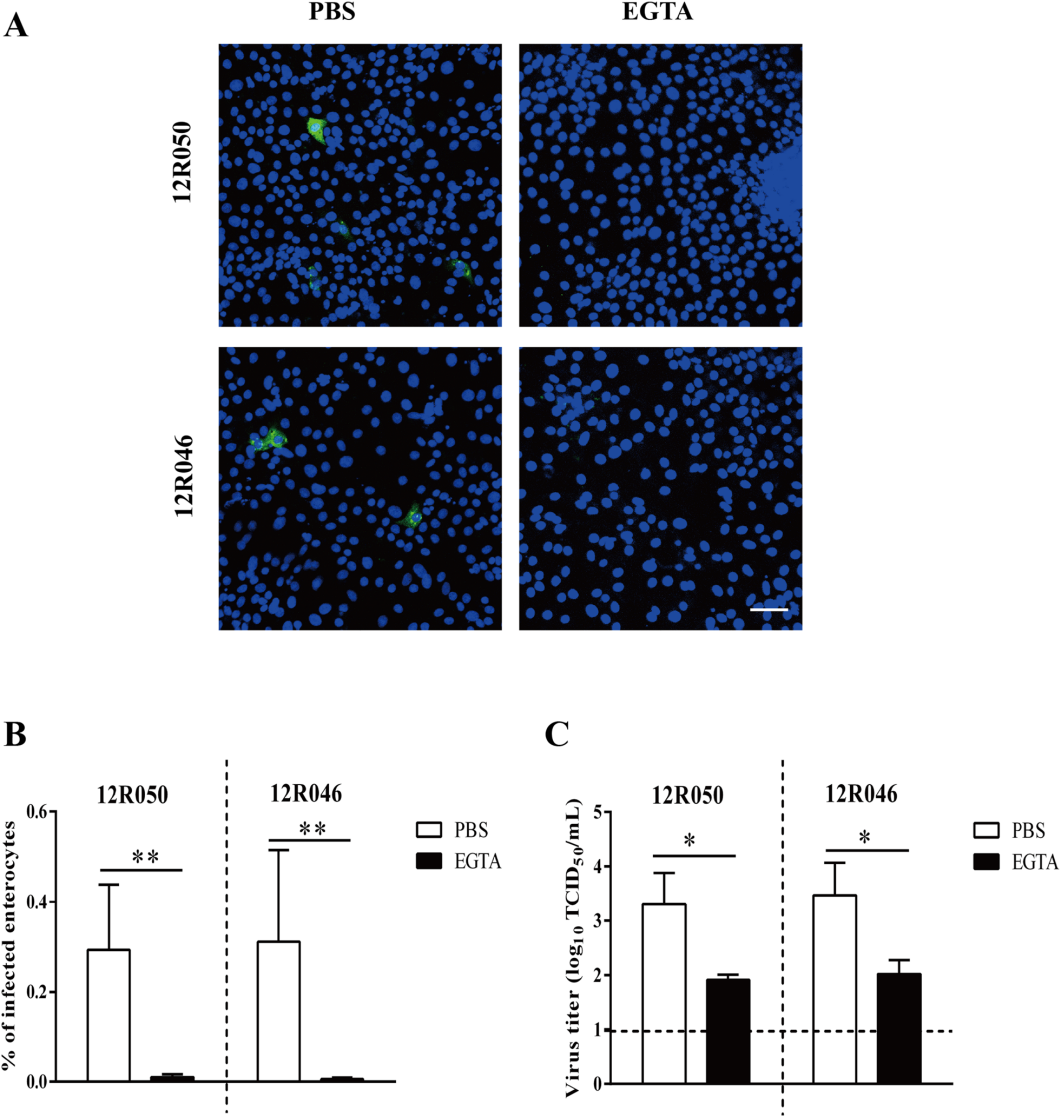
**EGTA treatment inhibits the internalization of rotavirus in enterocytes.** Cells were inoculated with rotavirus at 4 °C for 1 h. After removing the unbound viral particles, cells were treated with EGTA or PBS at 37 °C for 30 min. Then, the cells were further incubated for 9 h after 3 washings with DMEM at 37 °C. **A** Representative confocal images of infection in enterocytes. Scale bar: 50 µm. The percentage of infected cells (**B**) and virus titer (**C**) were determined. Data are expressed as the mean ± SD of the results of three separate experiments. Statistically significant differences are indicated with one asterisk (*p* < 0.05) and two asterisks (*p* < 0.01).

Next, we investigated whether EGTA is able to affect the internalization stage of rotavirus infection. After inoculation with rotavirus 12R050 and 12R046 (MOI of 0.1) at 4 °C for 1 h, enterocytes were treated with EGTA for 30 min. Cells were further cultured for 9 h and the percentage of infection and virus titer were assessed. As shown in Figure 5, after EGTA treatment, the percentage of infection was 29 and 50 times lower than after mock PBS treatment for 12R050 and 12R046, respectively. The virus titer after EGTA treatment (10^1.9^ ^± 0.1^ TCID_50_/mL for 12R050 and 10^2.0^ ^± 0.3^ TCID_50_/mL for 12R046) was lower than after mock PBS treatment (10^3.3 ± 0.6^ TCID_50_/mL for 12R050 and 10^3.5^ ^± 0.6^ TCID_50_/mL for 12R046).

**Figure 6:**
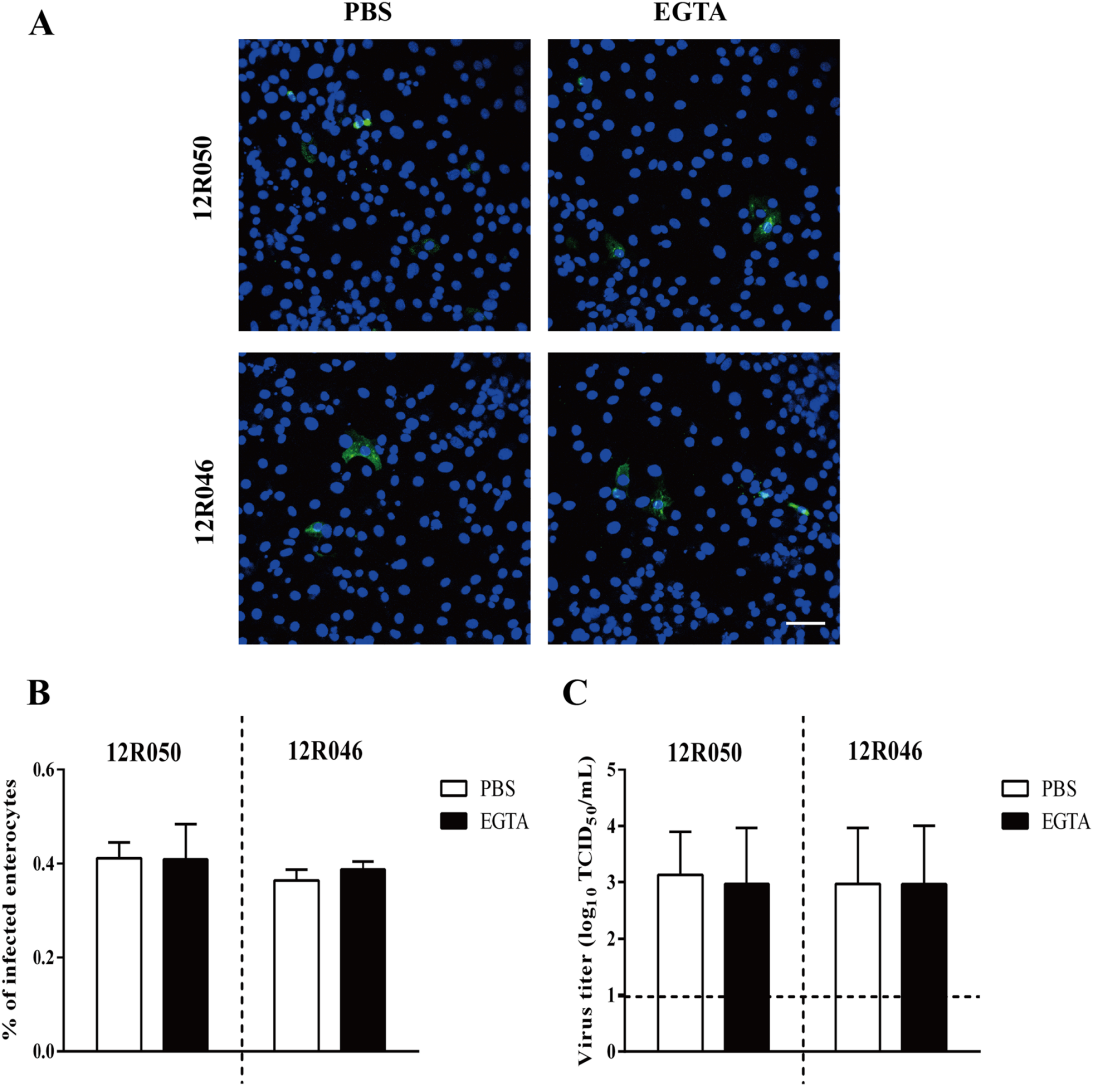
**EGTA treatment has no effect on the post-internalization stage of rotavirus particle in enterocytes.** Cells were inoculated with rotavirus at 37 °C for 1 h. After 3 washings, cells were treated with EGTA or PBS for 30 min. Then, the cells were further incubated for 9 h at 37 °C. **A** Representative confocal image of infection in enterocytes. Scale bar: 50 µm. The percentage of infected cells (**B**) and virus titer (**C**) were determined. Data are expressed as the mean ± SD of the results of three separate experiment.

Finally, we examined the effects of EGTA on the post-internalization stage of the rotavirus replication cycle. After inoculation with rotavirus 12R050 and 12R046 (MOI of 0.1) at 37 °C for 1 h, enterocytes were treated with EGTA for 30 min. The percentage of infected cells and the virus titer were determined at 9 hpi. After EGTA treatment, the percentage of infection was 0.41 ± 0.07% and 0.39 ± 0.02% and the virus titer was 10^3.0 ± 1^ TCID_50_/mL and 10^3.0 ± 1^ TCID_50_/mL for 12R050 and 12R046, respectively. After the mock treatment with PBS, the percentage of infection was 0.41 ± 0.03% and 0.36 ± 0.02% and the virus titer was 10^3.1 ±^ ^0.8^ TCID_50_/mL and 10^3.0 ±^ ^1^ TCID_50_/mL for 12R050 and 12R046, respectively (Figure 6).

### Effect of NA treatment of enterocytes on rotavirus replication

**Figure 7:**
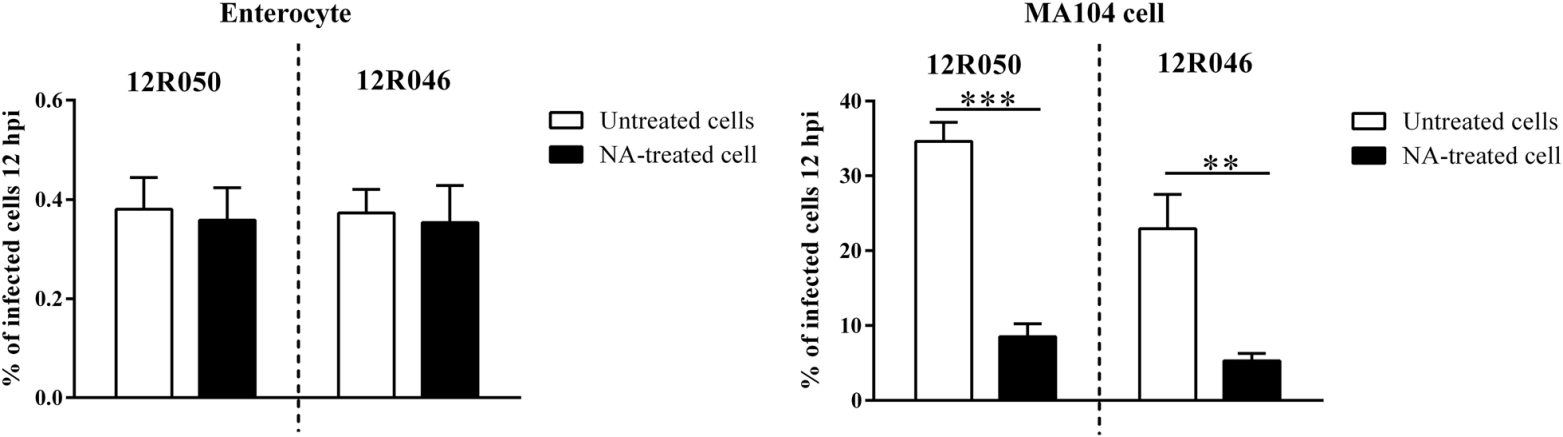
**Effect of neuraminidase treatment on the replication of rotaviruses in primary enterocytes and MA104 cells.** Primary enterocytes and MA104 Cells were treated with 50 mU/mL NA from *Vibrio Cholerae* for 1 h at 37 °C before inoculation with rotavirus at a MOI of 0.1. The percentage of infection was measured by immunofluorescence staining at 12 hpi. Data are expressed as the mean ± SD of the results of three separate experiments. Statistically significant differences are indicated with two asterisks (*p* < 0.01) and three asterisks (*p* < 0.001).

To assess the role of SAs on rotavirus replication in enterocytes, enterocytes were pretreated with 50 mU/mL NA prior to inoculation with rotavirus 12R050 and 12R046 (MOI of 0.1). The percentage of infection of the NA treated enterocytes was 0.36 ± 0.07% and 0.35 ± 0.07% for 12R050 and 12R046, respectively. The percentage of infection for mock treated enterocytes was similar as NA treated enterocytes (0.38 ± 0.06% and 0.37 ± 0.05% for 12R050 and 12R046, respectively). As a control, the percentage of infection of NA treated MA104 cells (8.5 ± 1.7% and 5.3 ± 1.0% for 12R050 and 12R046, respectively) was significantly lower than mock treated MA104 cells (34.6 ± 2.5% and 23.0 ± 4.6% for 12R050 and 12R046, respectively) (Figure 7).

## Discussion

Rotavirus infection is primarily restricted to the polarized epithelial cells of small intestines. Previous studies examining the polarity of rotavirus infections have been conducted in continuous cell lines and conflicting results were reported. In this study, we used primary enterocytes to explore the infection of rotavirus in polarized target cells, which is closely resembling the in vivo situation. Primary enterocytes formed a polarized epithelium with a stable TEER of 500–700 Ω cm^−2^ after 2 days of cultivation. We observed that treatment with EGTA disrupted the intercellular junctions of enterocytes leading to a significantly drop of the TEER to baseline levels and significantly enhanced the infection of rotavirus in enterocytes. EGTA was found to mainly affect the binding of rotavirus to enterocytes. Upon different inoculation route, rotavirus mainly infected enterocytes at the basolateral surface. To our knowledge, this study is the first to disrupt intercellular bridges in a primary enterocyte culture system to explore the infection of rotavirus in polarized primary enterocytes.

The polarized epithelium of the gastrointestinal system displays the highest turnover of all solid organs, undergoing complete renewal every 2 to 3 days in pigs. It maintains a constant and effective barrier function to restrict the movement of solutes, ions, macromolecules and pathogens across the mucosa. This regulation can be attributed to the junctional complex between adjacent epithelial cells which is composed of the tight junction (TJ), adherens junction (AJ) and desmosomes. TJs are located at the apicolateral borders of adjacent epithelial cells, and form belt-like areas responsible for selectively regulating the passage of ions and neutral molecules through the paracellular space. One of the most important functions of TJs is to restrict pathogen access to the basaloteral side of the epithelium and the systemic blood circulation. Paradoxically, some viruses utilize TJ proteins as receptors. Coxsackie B virus (CVB) and adenovirus bind to the coxsackie virus and adenovirus receptor (CAR) which is expressed at the TJ and is associated with ZO-1 protein [43]. Reoviruses bind to the junctional adhesion molecule (JAM) which is a TJ-localized integral membrane protein and JAM-A functions as coreceptor for rotavirus entry into MA104 cells [15, 20]. The TJ proteins occludin and claudin-1 facilitate the entry of hepatitis C virus (HCV) [44, 45]. Calcium is the most important ion in regulating intercellular junction integrity. Low level of calcium disrupts AJ of epithelial cells, which is supposed to be caused by removing calcium from binding sites on E-cadherin extracellular domains, leading to a conformational change and disruption of cell–cell adhesion [46, 47]. Depletion of extracellular calcium also mediates the redistribution of TJ proteins. Incubation of Caco-2 cells in calcium-free medium rapidly decreased transepithelial resistance and increased paracellular permeability, indicating a decreased tightness of TJ [48]. EGTA is a chelating agent which specifically sequestrates extracellular calcium. EGTA-induced depletion of calcium from the culture of polarized epithelial cells results in rapid splitting of ICJ. In our study, the primary enterocytes rapidly lost their ICJ after treatment with EGTA and became round, indicating that EGTA efficiently destroyed the ICJ of primary enterocytes. The disrupting effect of EGTA can be reversed by replenishing extracellular calcium [49]. In the present study, the enterocytes lost their contacts and became individual round cells after the EGTA treatment. The EGTA treated enterocytes reformed monolayers and maintained their viability after re-supplementation with calcium containing medium.

Destruction of the epithelial integrity by EGTA breaks down the barrier function of the epithelium against viruses. Van Cleemput et al. demonstrated that disruption of ICJ in equine respiratory mucosal explants and equine respiratory epithelial cells by EGTA significantly increased the infection of equine herpesvirus type 1 (EHV1) [29]. A similar effect of EGTA was found for bovine herpesvirus 4 (BoHV-4) [30]. Treatment of bovine nasal mucosal and tracheal explants and bovine respiratory epithelial cells with EGTA enhanced BoHV-4 infection [30]. Rotavirus is one of the major enteric viruses that replicates in intestinal epithelial cells, which seriously causes blunting of intestinal villi and enterocyte vacuolization and lysis. We investigated the effect of disruption of ICJ of enterocytes in a monolayer on rotavirus infection. Treatment of cultured enterocytes with EGTA significantly increased the virus replication, which confirmed that the intercellular junctions protect intestinal epithelial cells to rotavirus infection and that depletion of intercellular junctions can significantly boost rotavirus replication in intestinal epithelial cells. Furthermore, we found that EGTA mainly affected the binding of rotavirus to enterocytes. This indicates that a main receptor is present in the intercellular region. Further work will focus on the identification of this receptor. Previous work in the rotavirus field demonstrated that sialidase-sensitive animal rotavirus strains can use terminal sialic acid as cellular receptor, whereas sialidase-insensitive human rotavirus strains bind to glycans with internal sialic acid, such as GM1 [50]. Recently, more and more data suggest that human rotavirus strains specifically recognize A-type histo-blood group antigens (HBGAs) [12, 51]. The rotavirus strains used in this study are porcine rotaviruses with G5P[7] and G9P[23] genotypes [6]. According to the phylogenetic analyses done by Liu et al., P[7] and P[23] genotypes of porcine rotaviruses belong to the P[I] genogroup and should be considered to be sialidase-sensitive [51]. In our study, we demonstrated that by using MA104 cells, these rotavirus strains are sialidase-sensitive, because removal of the sialic acids of MA104 cells by treating the cells with neuraminidase significantly reduced viral replication. However, in primary porcine enterocytes, these low-passage P [7] and P[23] rotaviruses are sialidase-insensitive strains. The treatment of epithelial cells with neuraminidase did not affect viral replication, which indicated that these types of rotavirus strains do not bind terminal sialic acids on true target epithelial cells. These results demonstrate that for studying the receptors of rotaviruses, primary enterocytes should be used and not MA104 cells. We hypothesize that intercellular receptors of rotavirus are hidden by the intercellular junctions and that breaking down the intercellular junctions facilitates the exposure of this receptor and promotes rotavirus infection. Further investigations are needed to find out which proteins/glycans are these unknown receptors. The outer layer of the rotavirus contains calcium which stabilizes the trimeric VP7 [52]. During cell entry, the rotavirus triple layered particle uncoats, losing VP4 and VP7 [53]. In vitro, calcium chelation triggers uncoating of the triple layer particle. Loss of calcium causes VP7 to dissociate from the double layer particle and to release VP5* which is free to fold back to its most stable, post cleavage conformation to facilitate the membrane-disruptive step [54, 55]. Expressed VP7 protein undergoes an antigenic change upon calcium chelation, losing its ability to bind neutralizing monoclonal antibodies [52]. Abdelhakim et al. indicated that when the BSC-1 cells were pulsed with EDTA-containing medium, rotavirus does not internalize due to the loss of both VP7 and VP4, which significantly reduces rotavirus infection [56]. Similar results were observed with our primary enterocytes. We treated the cells with EGTA after binding of the rotavirus to investigate the effect of EGTA on the internalization stage of rotavirus. The results showed that calcium chelation at the internalization stage significantly blocked rotavirus infection. These findings indicate that rotavirus replication inside cells is mediated in a calcium-dependent manner.

Since the disruption of the intestinal epithelial cells’ integrity leads to enhanced rotavirus binding, we hypothesized that its primary binding/entry receptor is located at the basolateral surface of enterocytes. Therefore, we inoculated the primary enterocytes at either the apical or the basolateral surface. We found that both genotypes of rotavirus strains preferentially infected cells at the basolateral surface. Previous work that addressed the polarity of rotavirus infection in epithelial cells reported conflicting results. Svensson et al. found that the NA-sensitive rhesus rotavirus (RRV) infected Caco-2 cells in an asymmetric manner [35]. Ciarlet et al. demonstrated that NA-sensitive rotaviruses preferentially entered cells at the apical surface, while the NA-resistant rotaviruses entered cells at both the apical and basolateral surface [36]. Cevallos et al. reported that NA-sensitive rotaviruses infected IPEC-J2 cells more efficiently at the basolateral surface [38]. These conflicting results were conducted with different cell lines. In the present study, we demonstrated that rotavirus 12R050 and 12R046 are NA-sensitive for MA104 cell infections, while they are NA-resistant for enterocyte infections. Based on these results, we recommend investigating the polarity of rotavirus infections in the real target cell (primary enterocytes) and not in continuous cell lines. Taken together, these findings suggest that rotavirus mainly uses a basolateral receptor for infection of enterocytes. To our knowledge, our study is the first to use the primary enterocytes closely resembling the in vivo situation to demonstrate that porcine rotavirus strains are preferentially infecting via the basolateral domain of intestinal epithelial cells. Of course, more strains should be included to make a more general statement.

In conclusion, the present study demonstrates that the integrity of intestinal epithelium protects the epithelial cells against rotavirus infection. Disruption of the intercellular junctions by EGTA facilitates the binding of rotavirus to its receptor and increases the replication of rotavirus in enterocytes. Our study provides new insights on the replication of rotavirus in their target cells, which can create opportunities to develop effective strategies against rotavirus infections.
